# Cultural competency training of GP Registrars-exploring the views of GP Supervisors

**DOI:** 10.1186/s12939-015-0226-3

**Published:** 2015-10-06

**Authors:** Kelly Watt, Penny Abbott, Jenny Reath

**Affiliations:** School of Medicine-Campbelltown Campus Building 30.3.24, University of Western Sydney, Locked Bag 1797, Penrith, NSW 2751 Australia

**Keywords:** Cultural competency, Cultural competency education, Graduate medical education, General practice

## Abstract

**Introduction:**

An equitable multicultural society requires General Practitioners (GPs) to be proficient in providing health care to patients from diverse backgrounds. This requires a certain set of attitudes, knowledge and skills known as cultural competence. While training in cultural competence is an important part of the Australian GP Registrar training curriculum, it is unclear who provides this training apart from in Aboriginal and Torres Strait Islander training posts. The majority of Australian GP Registrar training takes place in a workplace setting facilitated by the GP Supervisor. In view of the central role of GP Supervisors, their views on culturally competent practice, and their role in its development in Registrars, are important to ascertain.

**Methods:**

We conducted 14 semi-structured interviews with GP Supervisors. These were audiotaped, transcribed verbatim and thematically analyzed using an iterative approach.

**Results:**

The Supervisors interviewed frequently viewed cultural competence as adequately covered by using patient-centered approaches. The Supervisor role in promoting cultural competence of Registrars was affirmed, though training was noted to occur opportunistically and focused largely on patient-centered care rather than health disparities.

**Conclusion:**

Formal training for both Registrars and Supervisors may be beneficial not only to develop a deeper understanding of cultural competence and its relevance to practice but also to promote more consistency in training from Supervisors in the area, particularly with respect to self-reflection, non-conscious bias and utilizing appropriate cultural knowledge without stereotyping and assumption-making.

## Introduction

In a multicultural society such as Australia, our health care system and health care practitioners must recognize and respect the needs of an increasingly diverse population. Cultural competence can increase equity of access and address social injustices through promoting recognition and acceptance of the impact of cultural differences, racism and discrimination on health and health care [1, 2]. The community context of General Practice (GP) means it is important that GP Registrars develop cultural competency during their training.

Cultural competence is defined as “a set of consistent behaviours, attitudes and policies that enable a system, agency or individual to work within a cross-cultural context or situation effectively” [3]. It encompasses more than an awareness of cultural differences in that the focus is on capacity to improve health and wellbeing through the integration of culture into the health service delivery [3]. Attributes of cultural competency include valuing diversity; having capacity for self-reflection and assessment, consciousness of the dynamics of cross-cultural interactions; and integration of cultural knowledge into clinical practice [3].

Studies exploring cultural competency curricula in both medical school and post-graduate settings have shown there is a large component of informal learning [4–6]. In many cases, there is a lack of training and students without the time and guidance to learn best practice skills develop coping and pragmatic skills instead [4], or become frustrated with the inadequate standard of health care they felt they are providing [7]. The influence that institutional culture and structure play in learning, (‘the hidden curriculum’), has been noted to affect outcomes of training with respect to cultural competency [5, 8]. Thus the views, skills and motivation of medical trainers to facilitate cross-cultural learning need to be understood.

In Australia, vocational general practice training is delivered through Regional Training Providers (RTPs), who provide mentorship and workshop based training to GP Registrars enrolled in the GP training program [9]. At least 2 years of their training is undertaken in the community, in GP placements of 6–12 months, supervised by experienced GP Supervisors.

Registrar learning about cultural competency remains unclear despite this being a core curriculum component [10–12] and a key expectation of the community. Currently the only mandated and minimum formal cultural training is in Aboriginal health, where GP Registrars are required to participate in cultural training [13].

Previous research has suggested that GP Supervisors may not consider cultural competency or addressing barriers to health care to be teaching priorities when training GP Registrars who are consulting specifically with Aboriginal and Torres Strait Islander people [14]. Since the majority of GP Registrar training is facilitated by the GP Supervisor, greater clarification of the Supervisor’s role in developing generic cultural competence skills of Registrars is needed [15]. In this study we aim to explore GP Supervisor views and report on our findings regarding their understanding of culturally competent practice and their role in developing Registrar cultural competence.

## Methods

### Setting

WentWest RTP provides GP Registrar training in the greater Western Sydney region [16]. This culturally diverse region has a population of approximately 750,000 people whose primary health care is provided by 293 General Practices [17]. In some parts of Western Sydney, up to half the residents were born overseas and up to 16 % of people have poor English proficiency [17]. Western Sydney also has the largest urban Aboriginal population in Australia, in some areas comprising around 4 % of the population [17].

### Participants

All WentWest Supervisors (120 in total) were invited to participate through email, and face to face at a routine Supervisor training workshop. Additionally, in a snowballing approach [18], six Supervisors were purposively approached to optimize the breadth of data collected. Sixteen Supervisors expressed interest in participating and all but one could then be contacted for interviews. One Supervisor expressing interest later declined participation due to other time commitments. Individual interviews were chosen as the best method for obtaining an in-depth understanding of the participants perspective [19]. Semi-structured interviews, via phone or in person, were audiotaped and transcribed after informed written participant consent was given. During the interviews participants were asked about their views and experiences with respect to culturally competent practice, their perception of its relevance to General Practice and registrar training, cross-cultural training in General Practice and their role in providing cross-cultural training to registrars. Questions regarding Supervisor demographics and perceived diversity of practice were also asked (See Table 1). All identifying information was removed from the transcripts and replaced with a code known only to the principal researcher who conducted the interviews. In this way, confidentiality of all participants was preserved throughout the analysis. All participants were offered a review of the transcript for checking, but this was declined in all cases. Participants were given honorarium payments of $100 for their time.

**Table 1:** Demographics of Supervisor participants

	Percentage (number)
Gender	
Male	50 % (7/14)
Female	50 (7/14)
Years of supervision experience	
<5 years	36 % (5/14)
5–10 years	36 % (5/14)
>10 years	28 % (4/14)
Place of vocational training	
Australia	64 % (9/14)
Overseas	36 % (5/14)
Languages spoken other than English	
None	36 % (5/14)
1	36 % (5/14)
2 or more	28 % (4/14)
Percentage of patients from a culturally diverse background as perceived by Supervisor	
<5	14 % (2/14)
5–25 %	36 % (5/14)
25–50 %	43 % (6/14)

### Analysis

Interview transcripts were analyzed thematically, with data managed using QSR International’s NVivo 10 software. Initial line-by-line coding was undertaken (KW), and focused codes were then explored in the rest of the data. Case-based, reflexive and conceptual memos were noted throughout and an audit trail was kept to enable confirmation [20]. A second investigator (PA) did thematic analysis independently and the resulting themes were discussed and compared in an iterative approach. Rival explanations were sought and negative case analysis improved credibility [20]. Focused themes reached saturation in the interview data.

The experience of the investigators as Registrar and GP Supervisor enhanced their understanding during the interviews as both had experience in learning and teaching and the current GP training process. The effect these roles may have had on data collection and interpretation of final results was also carefully reflected upon [18]. Throughout the data collection, several cultural mentoring sessions were held with an experienced Aboriginal cultural mentor [21]. This assisted to gain a deeper understanding of the developing concepts and themes throughout the analysis and provided peer debriefing from another perspective [20].

Ethics approval was provided by the University of Western Sydney Human Research Ethics Committee and the Aboriginal Health and Medical Research Council of NSW Ethics Committee. This study adheres to the RATS guidelines for reporting qualitative studies.

## Results

Fourteen GP Supervisors participated in the study. Their demographics are reported in Table 1. More than a third had undertaken GP training outside Australia and spoke at least one language other than English. Interviews ranged from 45 to 80 min duration with an average duration of 60 min. All Supervisor participants reported patient populations with less than 10 % Aboriginal and Torres Strait Islander patients. Half of the Supervisors reported more than 25 % of their patients were from culturally diverse backgrounds.

The resultant themes are presented in two broad sections; the views of GP Supervisors on culturally competent practice, and perceptions of their role in developing cultural competence in Registrars.

### Views on culturally competent practice

#### Understanding and practice

Most Supervisors were not familiar with the term ‘cultural competence‘, except for three who had broader roles in medical education. Supervisors described culture as a complex and dynamic entity that had an inescapable impact on health and health care as it shaped health beliefs and expectations. However, their views on how culture impacted on health care and the approach they took to their practice and teaching varied. Two different approaches were evident. While the approaches were not considered to be mutually exclusive, Supervisors appeared to fit with one or other approach in their day-to-day practice and perceptions about the relevance and importance of cultural competence in their practice. In the first approach Supervisors relied on interpersonal interactions within the consultation, exploring and responding to patients’ expressed needs, rather than adjusting their practice according to prior knowledge of their patients’ cultural background. Supervisors using the second approach incorporated prior knowledge of the patient’s cultural background and altered their approach accordingly. Both approaches required flexibility, non-judgmental attitudes and motivation on the part of the GP.

#### Exploring culture through interpersonal doctor-patient interactions

The majority of Supervisors (9 out of 14) perceived culture as just one of many important factors that impact on the consultation and therefore the patient’s health care, and did not believe it was necessary to have substantial background knowledge of each patient’s culture. They preferred to explore and respond to the individual’s expressed needs in the consultation, believing that treating a patient based on prior knowledge of cultural differences amounted to stereotyping and risked making assumptions.

GP1-Well, you can’t take the culture out of the individual. It’s part of the individual. So if you get the individual stuff right, culture is encompassed in that. The trouble with starting to isolate cultures, as we do with Aboriginal and Torres Strait Islanders, is that you can make it something bigger than it should be. It’s, oh my gosh, this patient’s an Aboriginal and I’ve got to start treating them differently. And you forget to treat them as an individual and with respect and the way that you might the next patient.

Historical and societal factors such as colonization, oppression and war specific to different cultural groups were seen as of secondary importance to the individual experience.

GP1-A person who’s been in a concentration camp, a refugee camp, whose been brought up a white Anglo-Saxon Australian in an orphanage, they’ve all got experience of trauma, okay. So I guess this is my point to you, is that you can use generic skills for this stuff.

Some Supervisors highlighted the difficulty of learning about so many other cultures. Consequently they preferred to practice with a generic style of respect and empathy for the individual patient and explore culture only when they perceived it had become relevant to the consultation. Re-presentation for the same problem or non-adherence with medical advice were suggested as examples of triggers to reflection on the impact of culture on the health or health care of the individual.

GP2-I think you build a picture and an interaction with people over time. If you think, this is the fourth time they’ve come back to me with what is obviously a viral respiratory tract infection and I think I’ve explained pretty well each time what’s going on, and yet they still always come in on day two when you think you’ve told them there’s not much point coming until day ‘X’ whatever, and I think in that situation, sometimes it’s very helpful-and you might even just ask somebody, “Well, what were you hoping that I’d be able to do for you today?”

By focusing more on building trust and rapport with the patient in a therapeutic alliance, some Supervisors argued that a lack of understanding about a patient’s culture would be forgiven.

GP11-So treating, you know, Aboriginal Torres Strait Islanders with respect and courtesy, I think is the first thing, and then knowing those finer nuances of their culture, and their feelings towards people who are sick, people who have passed on, use of names and appropriate mourning time, going back to be with family. You know, those finer nuances help, but I mean you don’t have to know those specifically if you’re already being very sensitive and caring and considerate to their needs in the first place.

GP14-I found a lot of them, when you don’t understand and you explain that and they see that you are trying, they are really nice you know and they want to try and help you because they know you don’t think any less of them, and you are trying to understand what they are saying, so they want to give you the right information. I have never had anyone that swore at me for that, or was rude.

Supervisors described learning what they needed to know, including the individual’s cultural beliefs, expectations and illness models, over time and through repeated consultations, that is, with continuity of care.

In general the Supervisors using this approach relied on what the patient chose to reveal about their background and culture. For example, identification of Aboriginality was mostly viewed as the patient’s responsibility, and some Supervisors expressed surprise at the number of patients discovered to be Aboriginal and Torres Strait Islander once they started asking patients in order to fulfill requirements of practice accreditation.

GP1-Probably about five… we were surprised. They just don’t identify themselves unless you ask.

Some supervisors added that being motivated to learn about the patient as a person was vital.

They commented that many colleagues without the same motivation might never gain a deeper understanding of the patient’s cultural perspective with this approach.

Supervisors generally identified language differences as an important barrier to providing healthcare; however GPs adopting the interpersonal approach to culture tended to approach language differences in a pragmatic and ad hoc fashion, again relying on the patient’s expressed needs within the consultation rather than anticipating communication needs. Many Supervisors expressed ambivalence towards the use of formally trained interpreters, both phone and face-to-face. Mostly it was assumed that family members or friends who were present in the consultation were there to interpret on behalf of the patient, although the problems associated with this were acknowledged.

GP2-I think we probably all fall in to the trap of using an interpreter that’s a family member when it’s not always appropriate. It’s often not appropriate. But it’s expedient. And particularly for the acute presentation, it’s kind of the best way to go.

GP8-If like I say, I have patients, I can’t speak Indian, language, so usually the children come with them and how much the children, like, pass my message to them I don’t know. But in terms of when I ask them questions and check things, usually I have no problem with these things.

The complexity and weight of the consultation usually dictated whether a formally trained interpreter was arranged. Many Supervisors did not feel that training in use of interpreters was required; instead they emphasized interpersonal communication techniques, such as using short simple, sentences and avoiding jargon.

#### Integrating prior cultural understanding into practice

A smaller number of Supervisors reported they incorporated prior knowledge of the patient’s cultural background in their consultation approach and did not solely rely only on patient information conveyed during the consultation.

GP5-I understood that you have to change your approach, when you’re dealing with males, females, elderly, so that cultural awareness that comes into the consultation, I think it’s really important and sometimes you can’t use your usual strategies or approach that you would with another patient. It can be a conscious or a subconscious thing, isn’t it? Because once you start talking to a patient, then you realize, okay, this is not working. Change it or-because you would know that the patient’s cultural background, you approach it from the beginning.

The Supervisors using this approach also expressed concern at the risk of stereotyping and making false assumptions about patients, and emphasized the importance of being equitable and respectful.

Consistent with this approach, Supervisors identified that cultural groups could have particular health needs and risks and could hold different expectations towards doctors and health services. Sometimes health beliefs and customs could act as a significant barrier to accessing health care in general practice.

GP13-As soon as I see a patient, their background makes, kind of directs me, to what kind of questions I will be asking, history et cetera, because as you know a lot of people from different backgrounds have different habits and have different risk factors.

GP10-And there’s already barriers-there’s huge barriers in the psychological world in the Middle Eastern people, particularly the males. Um, so generally adult male depression in the Middle Eastern person, your first battle is to actually convince them they’ve got a problem, and it’s not-not somebody who said something about them three years ago, you know. Bit of an issue to start with.

Some patient-centered approaches promoted within Australian general practice, such as sharing power within the consultation, informed consent including discussion of risks of treatment, and approaches to decision-making and individual autonomy, at times needed to be carefully broached by Supervisors with the knowledge that these approaches may be at odds with the patient’s cultural expectations.

GP10-We talk to patients about the, um, well, what we used to call informed consent, but the pros and cons of treatment, you know, the-the side effects as well as the benefits. A lot of patients find that very confronting, they’re not used to that. Um, so dealing with that open discussion regarding the side effects of medicine is something that you’ve got to be a little aware of because it may actually turn the patient off taking the medicine. It could be so simple, the potential side effect. And they’re not used to that in a lot of cultures, they don’t talk about the negative side of medication. The doctor’s seen as a trust relationship.

GP13-I have come across consultations where I had to deliver certain bad news and the families were insisting on delivering this news, and ‘you can’t do that doctor’, ‘you can’t say that to them doctor’, and it became a conflict…Yeah, I don’t stop and tell them you do what you like, this is my legal responsibility, this is my ethical-which it is and it is totally my right to say that. But you tend to lose ground when you do this. But I really, remember, those people you do this or it‘s mainly because of their culture. So you try to understand their culture, try to identify and then try to change.

Anticipating culturally sensitive areas of health including gender sensitive topics, breaking bad news, and issues surrounding death and terminal illness was considered important. Further to this, the Supervisor’s cultural awareness could lead to negotiating and mediating patient expectations or adjusting their customary practice, around issues such as health screening, physical examination, investigations, scripts, appointment bookings and waiting times.

Some Supervisors actively set about building bridges by educating staff and patients to decrease the risk of cross cultural misunderstandings in their practice. They aimed to incorporate recognition of cultural differences into the practice as a system as opposed to responding to individual needs as a result of triggers or misunderstandings.

Supervisors considered knowledge of appropriate resources within the community to be important. This ranged from using multilingual and culturally diverse practice staff to help inform the management of different patients, to building partnerships with a range of community health care providers who provided culturally and linguistically appropriate care.

GP5-We have got a dietician, also allied health professionals in the area who speak the language. So what we’ve done is even if we’re looking at the podiatry or dieticians, especially dieticians with diabetic patients-we tend to refer them to specific ones we know speak the language.

Written information in different languages with appropriate descriptions and wording was also used, however Supervisors noted the difficulty of having specific resources for patients from such a large number of cultural and language groups.

### Registrar training and cultural competence

All Supervisors perceived patient-centered skills, communication skills and general consultation skills to be of utmost importance and sought to develop these skills in their Registrars through a variety of methods, but rarely focused on specific cross-cultural aspects of care. Supervisors described several barriers to facilitating cross-cultural supervision. There was generally ambivalence amongst Supervisors in this study towards formal cultural competence training, both for Registrars and Supervisors.

#### Opportunistic teaching versus creating opportunities for cross-cultural supervision

Cross-cultural skills were often seen as a product of generic patient-centered skills. Many Supervisors viewed the development of knowledge of culturally sensitive topics, historical factors and health beliefs as best gained through the experience of working day-to-day as a general practitioner.

GP1 - I mean, in our practice, Registrars are going to get exposure to comprehensive care, continuous care, and patient centered care, and the culture is an important thing to factor in but it’s probably a secondary factor.

Cross-cultural aspects of care addressed during supervision occurred mostly on an ad hoc basis, in response to Registrar difficulties with particular patient encounters or as a result of patient feedback where cultural differences were perceived by the Registrar or Supervisor to be impacting.

GP5-Is it a topic that Supervisors are actually approaching with Registrars? I think as it’s probably more accidental that it’s happened rather than consciously. And so I’m thinking that that is one way where Supervisors can also use it and be made aware.

Some Supervisors felt that by role-modeling empathic and respectful behaviours in day-to-day practice, their Registrars would learn to adopt the same behaviours.

However a few Supervisors, particularly those in practices with a high proportion of culturally diverse patients, appeared to have a more planned approach around cross-cultural teaching, creating opportunities to incorporate supervision of the cultural aspects of care. They exposed their Registrars to complex patient presentations, and ensured their Registrars sat in on consultations in languages other than English, or with interpreters, and did home visits. They described mediating potential misunderstandings and educating their Registrars about cultural issues either before or after they had occurred.

GP5-So I must say, the other thing I try and do, if there’s a patient that is like this little girl, who is pretty complex, and she comes regularly for bloods, so if I see that she’s booked in with the Registrar, and it’s going to be the first time, I would just give them the background. I run through, like these are the difficulties, these are the things you will need to focus on and saying, look, in this culture, it is quite the done thing, accepted.

#### Barriers to cross-cultural teaching and supervision

Supervisors commonly identified that Registrar attitudinal resistance was a barrier to their ability to promote cross-cultural skills development. Most Supervisors found it challenging to assist Registrars who had difficulty performing well in a cross-cultural context. Registrars were often seen as either having an innate ability to self-reflect and communicate in an empathic manner or not.

GP3-Ultimately it is the personality of the person, no matter where they have come from, if they are kind-you can never teach kindness to someone.

GP2-I think the ability to reflect is a key part of it and I’m not entirely convinced you can teach non-reflective people to be reflective.

Supervisors described feeling restricted in the feedback they were able to give because of their conflicting roles as trainer, assessor and employer and because providing feedback on qualities of a Registrar perceived to be innate, such as empathy, ability to create rapport, and self-reflection, risked alienating or offending the Registrar.

GP12-A lot of Registrars, particularly now because you can get a lot of mature-age students; they’re very defensive and really a lot of Registrars you can’t criticize them whatsoever and they think they’re very, very good, they’re very experienced even though their knowledge in general practice is zero. However, because they’re from training bodies you can’t really – you have to approach the situation more gently compared to someone else. I think it’s give and take. If you find your Registrar a bit defensive, then you just back off.

Some Supervisors also commented that it was often even more difficult to give critical feedback to international medical graduate Registrars who were perceived to have experienced many more challenges in training and in their personal lives.

GP3-Some of the international trained graduates have gone through so much-they have come here as refugees, like, they have just gone through so much in their lives, you are hesitant to add any more to their burden.

Given these challenges at the GP Supervisor level, some Supervisors recommended an enhanced role for the RTP and medical educators in assisting Registrars with poor cross-cultural consultation skills.

GP11-Whether I can add to that training, look no, I don’t think that-I mean you can’t be all things to all people. So I think that probably that would be something better handled by the RTP, I expect them to carry the mantle for that one.

#### Cultural competence training

All Supervisors agreed that Registrars should develop cultural competence and most felt that this was an important area of training that currently lacked focus. However, some queried the importance of providing formal cultural competence training to all Registrars. These Supervisors suggested this training was only relevant to Registrars intending to work in areas of high cultural diversity or specifically in Aboriginal and Torres Strait Islander health, and could compete with other curriculum priorities such as clinical management.

Standalone workshops were generally seen as an acceptable way to establish the basic principles of cross-cultural practice consistently across the Registrars.

GP2-So I think ultimately it probably comes as the first step back to the medical educators and the RTP because you’re setting a tone, and you know your Registrar’s going to go through at least three different practices and each of those practices will be unique. You can’t replicate a similar experience for every Registrar except within the setting of the RTP and the medical educators and the teaching that’s provided there. That’s the only place you can get absolute consistency.

Some Supervisors recommended migrant doctors as a valuable resource in this training. Many valued the current Aboriginal and Torres Strait Islander cultural awareness workshops as a way of promoting an understanding of historical and cultural aspects of care of Aboriginal and Torres Strait Islander patients. Other GPs expressed the view that the Registrar training focus on Aboriginal and Torres Strait Islander health was excessive and politically driven.

GP12-We need doctors who work hard to look after the people, not sweet talkers, not people who talk politically correct.

Furthermore, some Supervisors expressed concerns of stereotyping being inadvertently reinforced in clinicians who had attended the workshops.

GP13-From my experience with Aboriginal patients, what I saw from them was totally different from the workshops. You attend these workshops and then you sit with a patient and you expect this stereotype. It kind of limits you to what to expect and you feel that you have got it all when you don’t really have a clue about what’s happening with them and sometimes we approach this consultation with that attitude, I had attended workshops-I should know you, I know you back to front and front to back. But the reality is different.

The large majority of Supervisors agreed that the most important way for Registrars to develop cultural competence was through on-the-job exposure to patients. They felt Registrars would learn to adapt their interpersonal consultation skills in order to treat patients from diverse backgrounds through this exposure.

GP13-You need time; you need time to understand people. It’s time in experience, and time in knowledge, and time in being able to read them as well. And time to connect with them. So it can't be taught from an article of a lecture or a workshop or from sitting with a patient for the first consultation. It just takes time and different levels if you know what I mean.

Most Supervisors saw a significant role for themselves in developing aspects of cultural competence in their Registrar by virtue of their role in generally facilitating Registrars’ skills, despite many reflecting that they did not currently actively engage in this process. Some did not feel comfortable facilitating this development of their Registrars’ skills citing lack of experience with cultural diversity and lack of confidence managing Registrars with attitudinal resistance and poor ability to self-reflect.

## Discussion

### Patient-centered approach benefits and risks

This study provides insight into how GPs approach cross-cultural consultations, as well as how they provide teaching and supervision in cultural competence with Registrars. Many Supervisors approached culture as a component of the individual’s presentation and placed emphasis on exploring cultural factors they perceived to be relevant to the individual patient over time rather than integrating prior knowledge about a patient’s culture into their consultations and practice. Many Supervisors described a preference to using the first approach as it was seen as a way to protect against stereotyping, as well as a pragmatic response to the extensive cultural diversity of Western Sydney.

Approaching individuals according to their cultural background was perceived to risk making assumptions about individuals for many of the Supervisors who participated in this study. Fear of stereotyping at times overshadowed exploring cultural differences. It was also a barrier to acceptance of cultural competence training, which was often seen to exacerbate stereotyping by focusing on cultural differences rather than on the practical skills needed to safely explore differences.

Stereotyping is the cognitive process where a mental representation is formed in an individual’s mind containing their beliefs, expectations and knowledge about a particular human group [22]. That representation may be positive or negative but is generally an oversimplification that allows for efficient processing and storing of information. In spite of the practitioner being consciously aware of the stereotype, it can lead to biased behaviour because of the influence on interactions with those perceived to be from that group [22]. It is a recurring theme and major concern for clinicians and educators in studies concerning cross-cultural practice and training [6, 7].

However, relying solely on expressed cultural needs during interpersonal interactions risks failing to address wider societal influences that already exist between cultural groups, such as institutional racism, mistrust of mainstream services and socio-cultural determinants of health. Clinicians must gain an understanding of the difference between utilizing cultural knowledge and stereotyping. This will enable understandings of differences to be incorporated into practice as a means of addressing health and management disparities.

### The practice of self-reflection

Many Supervisors in this study described self-reflection as a method of improving their insight and approach to different patients. However this reflection tended to focus on interpersonal elements of the consultation, as seen in other studies [23]. In contrast, few Supervisors described cultural self-reflection, which more specifically reflects on the role of the clinician, their culture and place within society in the context of privilege and disadvantage and how this perpetuates inequities between cultural groups [24].

Moreover, reflection was mostly in response to triggers in which the outcome or patient health choices did not meet GP expectations. This requires recognition of that unexpected outcome. Patients may self-select GPs over time and patients who don’t perceive culturally safe care may not access the service and remain underserviced [25] leaving the GP unaware of repeated cultural misunderstandings. In vulnerable populations where negative experiences with health services, mistrust and poorer health outcomes are common, waiting for triggers in order to adapt to the patient cultural needs, may result in important opportunities for engagement with health services being missed. In contrast, integrating culture into the health service delivery requires cultural knowledge to be incorporated into practice, providing a potentially safer environment that respects and anticipates cultural needs [26]. Relying on expressed cultural needs of patients is also problematic due to the high rate of unvoiced agendas during consultations [27] compounded in cross-cultural consultations by the additional communication complexities and reduced mutual understanding [28].

Effective cross-cultural understanding has been achieved in some instances through long-term trusting therapeutic relationships solely through repeated interpersonal interactions [23]. This could be broadened to include not only adapting to patients over time, but also to the community within which the GP works. However, as many Supervisors in this study noted, this relies greatly on the motivation of the individual clinician to explore culture within their therapeutic relationship and to remain in the community long enough to build those relationships. The inverse care law, where those with the greatest health needs often receive the least services, applies to culturally diverse minority groups and therefore health disparities are likely to continue unless this is addressed by the health profession as a whole [29].

### Addressing systems

Supervisors who incorporate prior knowledge of cultural background and altering their approach accordingly also described proactively adapting both the health service and the focus and style of consultation. This was noted to require self-reflection and interpersonal skills with an emphasis on treating all patients with equal and individual respect. Use of this approach appeared to be associated with acknowledgement of systemic barriers to health and health care for different cultural groups and greater reflection on the position of the doctor and medical culture within that system. Some Supervisors took an active role, acting as educators and advocates for patients who experienced barriers to health care. This role often resulted in a systems or practice-wide approach such as anticipation of the need for interpreters, or involvement of bilingual and bicultural practice staff in the care of patients.

This study focuses only on cultural competence in the context of general practice. The importance of wider systemic and societal changes in reducing health disparities cannot be overstated. Even a highly culturally competent individual will be limited in their ability to provide appropriate health care within systems and according to policies that are not culturally competent [3, 30]. As a key entry point to health care services, general practitioners have an important role in this change [26].

### Teaching approaches

The Supervisor approach to their own cross-cultural consultations tended to be reflected in their teaching approach. Supervisors tended to focus on developing patient-centered, general communication and consultation skills in their Registrars, rather than actively focusing on cultural competence. Cross-cultural knowledge and skills training was mostly undertaken in an ad-hoc way, in response to triggers such as Registrar uncertainty in clinical scenarios, consistent with the majority of teaching encounters within the Supervisor-Registrar relationship [31]. Therefore it appeared that learning about cultural competence required not only Registrar exposure to diverse patients to create triggers but also recognition of those triggers by the Supervisor or Registrar.

Key features of cultural competency such as cultural self-reflectiveness & sensitivity were often seen by the Supervisors in this study as innate and difficult to foster and this may explain their lack of focus on this in training. Some Supervisors also perceived a lack of relevance of cultural competence to areas of General Practice they believed lacked cultural diversity. However common cross-cultural misunderstandings and culturally sensitive topics have been found to recur across a wide range of settings [32] and equitable health care requires that GPs have an understanding of these in order to provide appropriate health care and address these barriers to access.

### Recommendations

Several strategies supported by this research are likely to enhance GP training in cultural competence. There is a need for improved definition and support of the Supervisor role in proactively seeking opportunities to promote the cultural competence of Registrars. Ensuring Registrars receive exposure to culturally diverse patients throughout their training may improve their skills and synergistically improve health disparities [33, 34]. Finally, formal Supervisor and Registrar training may be an effective means of standardizing both Registrar and Supervisor learning in this area [35, 36]. This training would ideally include greater focus on understanding the roots of health disparities, and a greater understanding of utilizing cultural knowledge, with skills to address non-conscious biases and stereotyping. Training to develop Supervisor techniques for promoting self-reflection and for teaching resistant Registrars may also facilitate greater Registrar cultural competency.

### Limitations

Sampling may have favored those Supervisors with an interest in cross-cultural medicine and therefore the results may reflect the views of Supervisors with higher levels of cultural competence and higher motivation to focus on this area than the wider Supervisor population.

The position of the investigators as Registrar and Supervisor affiliated with an Aboriginal Medical Service and WentWest RTP may have influenced the way that Supervisors felt able to disclose sensitive or difficult points of views but on the other hand may have brought a deeper understanding to the data.

## Conclusions

In alignment with Supervisors’ own approaches to cross cultural consultations, there appears to be a strong GP Supervisor focus on training Registrars to provide individualized care but less focus on promoting cultural self-reflection and cultural competence. The training practice-based learning of cross-cultural skills generally requires Registrar exposure to diverse patients and awareness of cultural barriers to care in order to create triggers for learning, followed by recognition of the need for discussion of cross-cultural dynamics by the Supervisor.

Formal training for both Registrars and Supervisors may be beneficial, not only to develop a deeper understanding of cultural competence and its relevance to practice, but also to promote more consistency in learning.

## Abbreviations

GP: General practice
RACGP: Royal Australian college of general Practitioners
ACRRM: Australian college of rural and remote Medicine
IMG: International medical graduate
NHMRC: National health and medical research Council
RTP: Regional training provider

## References

[1] Nairn S, Hardy C, Parumal L, Williams GA (2004). Multicultural or anti-racist teaching in nurse education: a critical appraisal. Nurse Educ Today.

[2] Downing R, Kowal E (2011). A postcolonial analysis of indigenous cultural awareness training for health workers. Health Sociol Rev: J Health Section Sociological Assoc.

[3] NHMRC (2006). Cultural competency in health: a guide for policy, partnerships and participation.

[4] Park ER, Betancourt JR, Kim MK, Maina AW, Blumenthal D, Weissman JS (2005). Mixed messages: residents’ experiences learning cross-cultural care. Acad Med.

[5] Culhane-Pera KA, Like RC, Lebensohn-Chialvo P, Loewe R (2000). Multicultural curricula in family practice residencies. Fam Med.

[6] Kai J, Bridgewater R, Spencer J (2001). “Just think of TB and Asians”, that’s all I ever hear’: medical learners’ views about training to work in an ethnically diverse society. Med Educ.

[7] Pieper HO, MacFarlane A (2011). “Im worried about what I missed”: GP registrars’ views on learning needs to deliver effective healthcare to ethnically and culturally diverse patient populations. Educ Health.

[8] Ewen S, Mazel O, Knoche D (2012). Exposing the hidden curriculum influencing medical education on the health of Indigenous people in Australia and New Zealand: the role of the Critical Reflection Tool. Acad Med.

[9] General Practice Education and Training Limited (2013). Australian General Practice Handbook 2013.

[10] Royal Australian College of General Practitioners (2011). Royal Australian college of general practitioners. Multicultural Health.

[11] Royal Australian College of General Practitioners (2011). Royal Australian college of general practitioners curriculum for Australian general practice 2011. Aboriginal and Torres strait islander health curriculum statement.

[12] Australian College of Rural and Remote Medicine (2013). Primary curriculum fourth edition.

[13] O’Shea A, Royal Australian College of General Practitioners (2010). Cultural safety training: identification of cultural safety training needs. Cultural safety training.

[14] Abbott P, Reath J, Gordon E, Dave D, Harnden C, Hu W (2014). General practitioner supervisor assessment and teaching of registrars consulting with aboriginal patients-is cultural competence adequately considered?. BMC Med Educ.

[15] Mann KV (2011). Theoretical perspectives in medical education: past experience and future possibilities. Med Educ.

[16] Wentwest-Overview. [http://www.wentwest.com.au/registrars-medical-students/training-registrars] [Accessed 5 October 2015].

[17] Limited W (2010). Western Sydney population health and workforce needs assessment summary.

[18] Noy C (2008). Sampling knowledge: The hermeneutics of snowball sampling in qualitative research. Int J Soc Res Methodol.

[19] Hansen EC (2006). Successful qualitative health research: a practical introduction.

[20] Shenton AK (2004). Strategies for ensuring trustworthiness in qualitative research projects. Educ Inf.

[21] General Practice Education and Training Limited (2011). Guide to general practice training in aboriginal and Torres strait islander health.

[22] Stone J, Moskowitz GB (2011). Non-conscious bias in medical decision making: what can be done to reduce it?. Med Educ.

[23] Rosenberg E, Richard C, Lussier MT, Abdool SN (2006). Intercultural communication competence in family medicine: lessons from the field. Patient Educ Couns.

[24] Cultural Safety Training Standards Committee (2011). Creating the NACCHO cultural safety training standards and assessment process: a background paper.

[25] Pink BPA (2008). The health and welfare of Australia’s aboriginal and Torres strait islander peoples.

[26] National Health and Medical Research Council (2005). Increasing cultural competency for healthier living & environments.

[27] Barry CA, Bradley CP, Britten N, Stevenson FA, Barber N (2000). Patients’ unvoiced agendas in general practice consultations: qualitative study. BMJ.

[28] Harmsen H, Meeuwesen L, van Wieringen J, Bernsen R, Bruijnzeels M (2003). When cultures meet in general practice: intercultural differences between GPs and parents of child patients. Patient Educ Couns.

[29] Grabovschi C, Loignon C, Fortin M (2013). Mapping the concept of vulnerability related to health care disparities: a scoping review. BMC Health Serv Res.

[30] Weech-Maldonado R, Elliott M, Pradhan R, Schiller C, Hall A, Hays RD (2012). Can hospital cultural competency reduce disparities in patient experiences with care?. Med Care.

[31] Wearne S, Dornan T, Teunissen P, Skinner T (2012). General practitioners as supervisors in postgraduate clinical education: an integrative review. Med Educ.

[32] Betancourt JR, Cervantes MC (2009). Cross-cultural medical education in the United States: key principles and experiences. Kaohsiung J Med Sci.

[33] Paez KA, Allen JK, Carson KA, Cooper LA (2008). Provider and clinic cultural competence in a primary care setting. Soc Sci Med.

[34] Blane DN, Hesselgreaves H, McLean G, Lough M, Watt GC (2013). Attitudes towards health inequalities amongst GP trainers in Glasgow, and their ideas for changes in training. Educ Prim Care.

[35] Beach MC, Price EG, Gary TL, Robinson KA, Gozu A, Palacio A (2005). Cultural competence: a systematic review of health care provider educational interventions. Med Care.

[36] Lie DA, Lee-Rey E, Gomez A, Bereknyei S, Braddock CH (2011). Does cultural competency training of health professionals improve patient outcomes? A systematic review and proposed algorithm for future research. J Gen Intern Med.

